# Sodium bicarbonate supplementation prevents skilled tennis performance decline after a simulated match

**DOI:** 10.1186/1550-2783-7-33

**Published:** 2010-10-26

**Authors:** Ching-Lin Wu, Mu-Chin Shih, Chia-Cheng Yang, Ming-Hsiang Huang, Chen-Kang Chang

**Affiliations:** 1 Graduate Institute of Sports and Health Management, National Chung Hsing University, 250 Kuo Kuang Road, Taichung, 402, Taiwan; 2 Department of Laboratory Medicine, China Medical University and Hospital, 91 Hsueh-Shih Rd, Taichung, 404, Taiwan; 3 Department of Athletics, National Taiwan College of Physical Education, 16, Sec 1, Shuan-Shih Rd, Taichung, 404, Taiwan; 4 Sport Science Research Center, National Taiwan College of Physical Education, 16, Sec 1, Shuan-Shih Rd, Taichung, 404, Taiwan

## Abstract

The supplementation of sodium bicarbonate (NaHCO_3_) could increase performance or delay fatigue in intermittent high-intensity exercise. Prolonged tennis matches result in fatigue, which impairs skilled performance. The aim of this study was to investigate the effect of NaHCO_3 _supplementation on skilled tennis performance after a simulated match. Nine male college tennis players were recruited for this randomized cross-over, placebo-controlled, double-blind study. The participants consumed NaHCO_3 _(0.3 g. kg^-1^) or NaCl (0.209 g. kg^-1^) before the trial. An additional supplementation of 0.1 g. kg^-1 ^NaHCO_3 _or 0.07 g. kg^-1 ^NaCl was ingested after the third game in the simulated match. The Loughborough Tennis Skill Test was performed before and after the simulated match. Post-match [HCO_3_^-^] and base excess were significantly higher in the bicarbonate trial than those in the placebo trial. Blood [lactate] was significantly increased in the placebo (pre: 1.22 ± 0.54; post: 2.17 ± 1.46 mM) and bicarbonate (pre: 1.23 ± 0.41; post: 3.21 ± 1.89 mM) trials. The match-induced change in blood [lactate] was significantly higher in the bicarbonate trial. Blood pH remained unchanged in the placebo trial (pre: 7.37 ± 0.32; post: 7.37 ± 0.14) but was significantly increased in the bicarbonate trial (pre: 7.37 ± 0.26; post: 7.45 ± 0.63), indicating a more alkaline environment. The service and forehand ground stroke consistency scores were declined significantly after the simulated match in the placebo trial, while they were maintained in the bicarbonate trial. The match-induced declines in the consistency scores were significantly larger in the placebo trial than those in the bicarbonate trial. This study suggested that NaHCO_3 _supplementation could prevent the decline in skilled tennis performance after a simulated match.

## Introduction

Tennis is an intermittent sport with the actual playing time being 17-28% of total match duration [1]. The remainder of the time is recovery between points and games. On average, the rallies last 4.3-7.7 sec in men's Grand Slam tournament matches [2]. At the stroke frequency of approximately 0.75 shots. sec^-1 ^[2], the cumulative effect of the repetitive short-term high-intensity efforts throughout prolonged tennis matches could result in significant neuromuscular fatigue [1,3], which in turn may impair certain aspects of skilled performance [4,5]. Indeed, the stroke accuracy was significantly decreased in competitive tennis players near the point of volitional fatigue [6]. Stroke accuracy and velocity were also significantly decreased after a strenuous training session (average rating of perceived exertion (RPE) 15.9/20) in well-trained tennis players [7].

One of the potential factors that may influence the skilled tennis performance is neural function. The central activation failure, changes in neurotransmitter levels and disturbance in excitation-contraction coupling have been suggested to play an important role in the development of fatigue in prolonged tennis matches [3,8]. The decline in maximal voluntary contraction and electromyographic activity of knee extensor muscles occurred progressively during a 3-hour tennis match, indicating a decreasing number of motor units that are voluntarily recruited [3]. The impairments in neural functions in lower limbs may lead to the slower acceleration in movement and the inability to reach the optimal stroke position. In addition, the neural impairments in forearm muscles may result in the poor control of the racquet.

Alkalinizing agents including sodium bicarbonate (NaHCO_3_) have been proposed as ergogenic aids for their potential effects on providing enhanced extracellular buffer capacity, leading to the elevated proton (H^+^) efflux from the contracting musculature [9,10]. The increased intramuscular [H^+^] during exercise has been considered as one of the major causes of muscle fatigue [11]. It has been suggested that H^+ ^accumulation would inhibit the enzymes involved in oxidative phosphorylation and glycolysis. It would also reduce Ca^2+ ^binding to troponin C and inhibit the sarcoplasmic reticulum enzyme Ca^2+^-ATPase [11,12]. Indeed, previous studies generally agreed that NaHCO_3 _supplementation was beneficial for the performance in a single bout of high-intensity exercise lasting 1-7 min [13,14], and intermittent short-term high-intensity exercise [15-17]. It has also been shown that NaHCO_3 _supplementation increased the total work output during a 1-hr competitive cycling [18]. Furthermore, NaHCO_3 _supplementation could improve total power output in a 30 min high-intensity intermittent cycling exercise representative of various ball games [19]. Nevertheless, several studies failed to find ergogenic effect of NaHCO_3 _supplementation on exhaustive short-term cycling [20] or resistance exercise [21].

Recently, the potential role of NaHCO_3 _supplementation in alleviating the exercise-induced impairment in the neural functions has been proposed. NaHCO_3 _supplementation has been shown to increase muscle fiber conduction velocity and reduce force decline in sustained maximal contraction after a 50-min submaximal cycling [22]. With the potential role of NaHCO_3 _in preserving the neural functions after prolonged exercise, we hypothesized that NaHCO_3 _supplementation may prevent the fatigue-induced decline in skilled tennis performance. The aim of this study was to investigate the effect of NaHCO_3 _supplementation on skilled tennis performance after a simulated match.

## Materials and methods

### Participants

Nine male Division I college tennis players (age 21.8 ± 2.4 years; height 1.73 ± 0.07 m) were recruited. All participants have competed in the national level. All participants were given their written informed consent. The study protocol was approved by the Human Subject Committee of National Taiwan College of Physical Education.

### Experimental design

**Figure 1:**
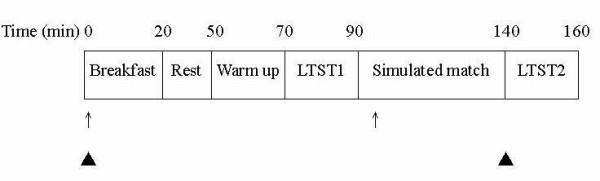
**Experimental design of the study**. LTST: Loughborough tennis skill test; ↑: NaHCO_3 _or placebo supplementation; (black triangle): blood sampling.

This study used a randomized cross-over, placebo-controlled, double-blind design. Each participant completed 2 experimental trials, bicarbonate and placebo, in a randomized order. The 2 trials were separated by 1 week. The schedule of dietary supplementation, exercise test, and blood sampling is shown in Figure 1. All trials were performed in the same outdoor tennis court with a hard surface. The temperature at the start of the exercise was 34.5 ± 3.2°C and 34.4 ± 3.4°C in the placebo and bicarbonate trial, respectively. The relative humidity was 47.5 ± 3.0% and 47.2 ± 3.6% in the placebo and bicarbonate trial, respectively. They were not significantly different between the trials. The participants familiarized with the test protocol and court in a training session 1 week before the experiment. The participants were instructed to maintain their training schedule and to consume exactly the same diet for 2 days before each trial. All participants were also asked to abstain from alcohol, caffeine, and tobacco consumption for 48 hours before each trial.

On the experimental days, the participants reported to the laboratory after an overnight fast. Body composition and body weight were measured using bioimpedance analysis method (InBody 3.0, Biospace, Seoul, Korea) before obtaining fasting blood samples. In the two trials, the participants had similar body weight (placebo: 67.90 ± 11.38 kg; bicarbonate: 68.04 ± 11.31 kg) and body fat (placebo: 16.11 ± 5.01%; bicarbonate: 15.48 ± 4.79%).

### Dietary protocol

After given fasting blood samples, the participants consumed NaHCO_3 _(0.3 g kg^-1 ^body mass) or placebo (NaCl, 0.209 g kg^-1^, equal amount of sodium) in 250 ml water. A standard breakfast (1.5 g. kg^-1 ^carbohydrate, including white bread, jam, and glucose drink) was ingested 20 min after the drink consumption. A 100 ml drink containing 0.1 g. kg^-1 ^NaHCO_3 _or 0.07 g. kg^-1 ^NaCl was ingested after the third game in the simulated match.

### Tennis skill test

The Loughborough Tennis Skill Test [4] was performed before and after the simulated match. Briefly, the test measured the accuracy and consistency of service and forehand and backhand ground stroke to both sides of the court. The players served 10 balls each at match pace from the right and left service area. The target was a 4.0 m × 0.6 m region marked at the end portion of the service box in the opposite court. Subsequently, the players performed forehand and backhand ground strokes cross-court and down the line with 10 balls each. The balls were fed by a ball serving machine (Tennis Tower Competitor, Sports Tutor Inc., Burbank, CA, USA) at the pace of 15 balls per min. A 1.5 m × 1.5 m target was placed in the rear corner of both singles court areas. The accuracy score was the number of balls which were landed on the designated target. The consistency score was the number of balls landed within the singles court on the designated side (excluding the target). The entire tests were recorded by a digital video camera for latter examination to ensure the accuracy of records. The on-site scoring and video analysis were performed by the same research personnel who were blind to the treatment.

### The simulated match

The simulated match consisted of 12 games, alternating receiving and service games. Each game consisted of 6 points and 6 balls were hit in each point. The balls were fed at the frequency of 6 balls/10 sec by a ball serving machine. The receiving games (game 1, 3, 5, 7, 9 and 11) started from a forehand ground stroke, followed by 2 backhand ground strokes, a forehand ground stroke, and 2 volleys. The service games (game 2, 4, 6, 8, 10 and 12) started from a service, followed by 2 backhand ground strokes, a forehand ground stroke, and 2 volleys. The participants were asked to return to the central line during the ground strokes, and to approach to the net during volleys. A 20 sec break was allowed between each point, and a 90 sec break was allowed after game 3, 5, 7, 9 and 11. The entire simulated match lasted approximately 50 min.

Heart rate was monitored throughout the study period using a short-ranged telemeter (EXEL SPORT, Cardiosport, West Sussex, UK). The RPE was recorded using the Borg scale before and after the skill tests and each game of the simulated match. Water was given *ad libitum *in the first trial, and the timing and amount of consumption were recorded. The same timing and amount of water consumption were repeated in the second trial. The average water consumption during the trials was 1089 ± 283 ml.

### Blood sampling and analysis

Blood samples were taken from a forearm vein by a trained nurse. The post-exercise blood samples were taken immediately after the simulated game. The needles were rinsed with 0.2% heparin before the sampling. A plastic seal was immediately applied to the syringe after blood collection to avoid the contact with the ambient air. The blood samples were put in ice bath and sent to the laboratory for analysis immediately.

Blood [lactate] was measured with a commercial kit (Roche Diagnostics, Indianapolis, IN, USA) using an autoanalyzer (Beckman SYNCHRON LX20 PRO, Fullerton, CA, USA). Blood [HCO_3_^-^], pH, hemoglobin, and base excess were analyzed using a blood gas analyzer (Synthesis 25, Instrumentation Laboratory, Lexington, MA, USA). Blood [lactate] and [HCO_3_^-^] were adjusted to the change in plasma volume [23].

### Statistical analysis

All values were expressed as means ± standard deviation. A two-way analysis of variance (ANOVA) with repeated measures was used to analyze the biochemical parameters and skill test scores. The independent variables included trial (bicarbonate and placebo) and time (before and after the simulated match). The trial × time interaction effect was used to test the null hypothesis of no difference in change over time between the 2 trials. When a significant main effect was found, the Ryan-Holm-Bonferroni step-wise method was used to determine the location of the variance [24]. The effect size of a variable was calculated with the following equation:

Effect size=|mean before the trial–mean after the trial|/standard deviation before the trial

The analysis was performed with SPSS 10.0. A P-value less than 0.05 was considered statistically significant.

## Results

**Figure 2:**
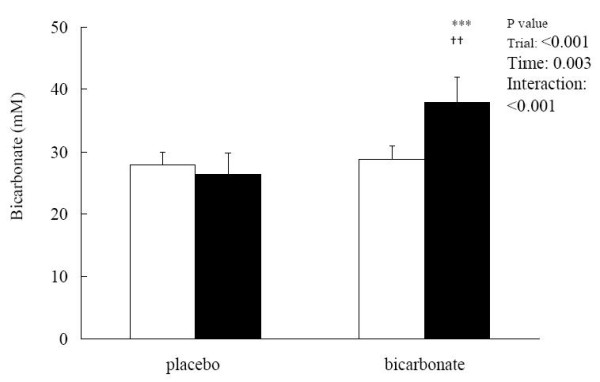
**Blood bicarbonate concentrations before (white square) and after (black square) the simulated match in placebo and bicarbonate trials**. ***p < 0.001, before vs after in the same trial; ^††^p < 0.01, bicarbonate vs placebo trial.

**Figure 3:**
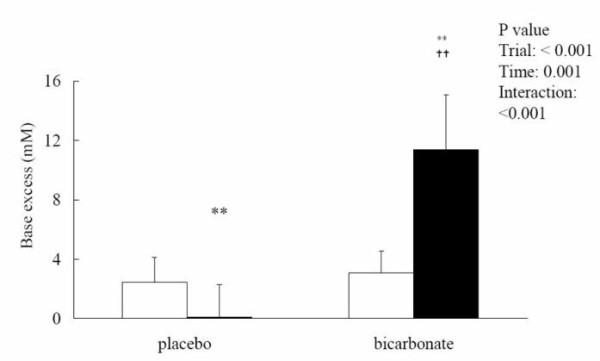
**Blood base excess before (white square) and after (black square) the simulated match in placebo and bicarbonate trials**. **p < 0.01, before vs after in the same trial; ^††^p < 0.01, bicarbonate vs placebo trial.

**Figure 4:**
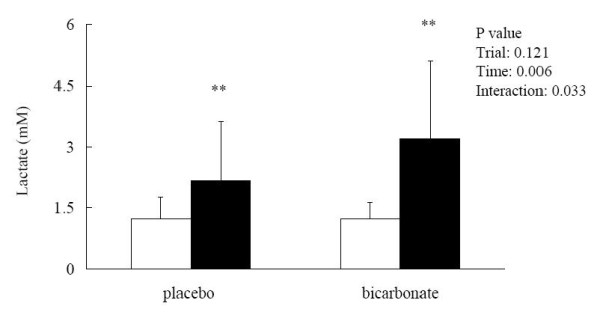
**Blood lactate concentrations before (white square) and after (black square) the simulated match in placebo and bicarbonate trials**. **p < 0.01, before vs after in the same trial.

**Figure 5:**
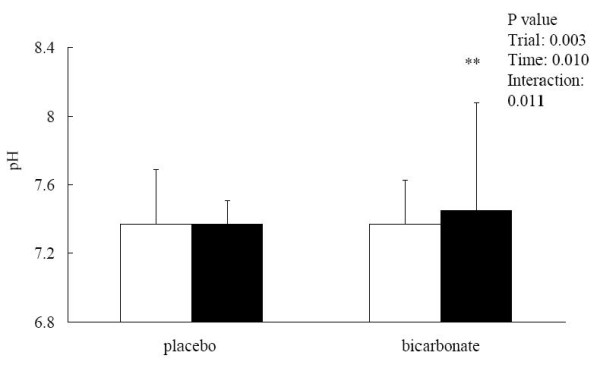
**Blood pH before (white square) and after (black square) the simulated match in placebo and bicarbonate trials**. **p < 0.01, before vs after in the same trial.

Blood [HCO_3_^-^] remained unchanged after the match in the placebo trial (pre: 27.99 ± 2.02; post: 26.37 ± 3.50 mM) but was significantly elevated in the bicarbonate trial (pre: 29.84 ± 2.16; post: 37.98 ± 3.15 mM, p < 0.05; effect size = 4.23) (Figure 2). The match-induced change in blood [HCO_3_^-^] was significantly different between the 2 trials (interaction effect p < 0.001; effect size = 2.92). Base excess showed opposite patterns between the 2 trials. The post-match base excess was significantly lower than the pre-match level in the placebo trial (pre: 2.46 ± 1.68; post: 0.12 ± 2.15 mM, p < 0.05; effect size = 1.39) but was significantly elevated in the bicarbonate trial (pre: 3.08 ± 1.47; post: 11.36 ± 3.70 mM, p < 0.05; effect size = 5.63) (Figure 3). Post-match [HCO_3_^-^] and base excess were significantly higher in the bicarbonate trial than those in the placebo trial. Blood [lactate] was significantly increased after the match in both placebo (pre: 1.22 ± 0.54; post: 2.17 ± 1.46 mM, p < 0.05; effect size = 1.76) and bicarbonate (pre: 1.23 ± 0.41; post: 3.21 ± 1.89 mM, p < 0.05; effect size = 4.83) trials (Figure 4). The match-induced change in blood [lactate] was significantly higher in the bicarbonate trial than that in the placebo trial (interaction effect p < 0.05; effect size = 1.73). Blood pH remained unchanged after the match in the placebo trial (pre: 7.37 ± 0.32; post: 7.37 ± 0.14, p > 0.05) but was significantly increased in the bicarbonate trial (pre: 7.37 ± 0.26; post: 7.45 ± 0.63, p < 0.05; effect size = 0.31) (Figure 5).

The accuracy and consistency scores of service and ground stroke in the Loughborough Tennis Skill Tests before and after the simulated match in both trials are presented in Table 1. The service consistency was significantly decreased after the simulated match in the placebo trial (95% confidence interval (CI) before: 12.7-21.1; after: 6.5-15.7; p < 0.05), but remained unchanged in the bicarbonate trial. The effect size for service consistency was 1.07 and 0.04 in the placebo and bicarbonate trial, respectively. The match-induced decline in service consistency was significantly larger in the placebo trial compared to that in the bicarbonate trial (interaction effect p = 0.004; effect size = 1.26). The 95% CI for the forehand ground stroke consistency before and after the placebo trial was 8.3-12.7 and 7.6-10.6, respectively. The 95% CI for the forehand ground stroke consistency before and after the bicarbonate trial was 6.8-9.2 and 7.3-11.3, respectively. The match-induced decline in forehand ground stroke consistency was also significantly larger in the placebo trial than that in the bicarbonate trial (interaction effect p = 0.046; effect size = 2.06).

**Table 1:** The consistency and accuracy scores of service and ground stroke before and after the simulated game in placebo and bicarbonate trials (mean ± standard deviation).

	Placebo		Bicarbonate		Main effect (P-value)		
	
	Before	After	Before	After	Trial	Time	Interaction
Service (out of 20)							

Accuracy	4.1 ± 1.8	4.5 ± 1.5	3.2 ± 2.6	3.8 ± 1.9	0.215	0.254	0.844

Consistency	16.9 ± 5.4	11.1 ± 6.0^†^	13.8 ± 5.1	13.6 ± 5.9	0.861	0.059	0.004**

Gs-Total^a ^(out of 40)							

Accuracy	5.5 ± 3.3	5.2 ± 2.5	6.0 ± 3.1	5.3 ± 2.2	0.758	0.446	0.694

Consistency	19.5 ± 4.2	17.1 ± 4.3	17.6 ± 2.8	19.0 ± 4.5	1.000	0.575	0.088

Gs-Forehand (out of 20)							

Accuracy	3.5 ± 1.5	2.7 ± 2.1	3.7 ± 1.9	2.3 ± 1.2	0.850	0.065	0.493

Consistency	10.5 ± 2.8	9.1 ± 2.0	8.0 ± 1.6	9.3 ± 2.6	0.237	0.943	0.046*

Gs-Backhand (out of 20)							

Accuracy	2.0 ± 2.1	2.3 ± 1.0	2.2 ± 1.8	1.8 ± 1.9	0.868	1.000	0.464

Consistency	9.4 ± 2.7	8.0 ± 2.5	9.7 ± 2.7	9.5 ± 3.0	0.391	0.046*	0.475

^a^GS: ground stroke; *p < 0.05, **p < 0.01; ^†^p < 0.05, before vs after in the same trial.

The average heart rate after each game in the simulated match was 173 ± 13 and 170 ± 20 beats. min^-1 ^in the placebo and bicarbonate trial, respectively (p > 0.05). The RPE after the simulated game was 15.7 ± 1.9 in the placebo trial and 15.2 ± 2.8 in the bicarbonate trial (p > 0.05).

The levels of hematocrit before and after the placebo trial were 44.8 ± 3.1 and 43.7 ± 2.6%, respectively. The levels before and after the bicarbonate trial were 45.7 ± 2.4 and 44.2 ± 2.2%, respectively. The match-induced changes in hematocrit were insignificant in both trials, indicating the adequate hydration status of the participants during the trials.

## Discussion

The results of this study suggested that NaHCO_3 _supplementation could prevent the decline in skilled tennis performance after a simulated match. The service and forehand ground stroke consistency was maintained after a simulated match in the bicarbonate trial. On the other hand, these consistency scores were decreased after the match in the placebo trial. Furthermore, in forehand and backhand ground strokes combined, the consistency showed a trend of decrease after the simulated match in the placebo trial (effect size = 0.57) while it increased slightly in the bicarbonate trial (effect size = 0.50) (interaction effect p = 0.088). To our knowledge, this is the first study that showed the effect of NaHCO_3 _supplementation on skilled performance in racquet sports.

Previous studies have focused on the effect of NaHCO_3 _on physical performance [14,18,25,26]. Only two studies investigated the effect of NaHCO_3 _supplementation on skilled sport performance [16,27]. It was reported that NaHCO_3 _supplementation could increase punch efficacy, the number of successful punches thrown and landed, by 5% in real boxing matches [27]. Another study revealed that NaHCO_3 _supplementation increased the number of judo-specific throws (*ippon seoi nague*) completed in the second and third round of a 3-round test. These authors contributed the effect of NaHCO_3 _supplementation to the enhanced extracelluar buffer capacity, lower intramuscular acidity, and increased strong ion difference which may affect Ca^2+ ^release in skeletal muscle [16,27]. Interestingly, these 2 studies also reported no effect of NaHCO_3 _supplementation on RPE, similar to our results. It suggested that NaHCO_3 _supplementation may increase skilled performance without the impact on psychological perception of fatigue.

In this study, blood [lactate] after the simulated match was 2.17 ± 1.46 and 3.21 ± 1.89 mM in the placebo and bicarbonate trial, respectively. The concentrations were similar to the previously reported results of 1.5-2.3 mM after real tennis match plays [28,29]. The induced alkalosis and increased post-match [lactate] in the bicarbonate trial were similar to the results in previous studies [15,19,30]. The significantly higher post-match [HCO_3_^-^] and base excess in the bicarbonate trial indicated enhanced extracellular buffer capacity. As the result, blood pH was significantly increased despite a significant increase in [lactate] after the simulated game in the bicarbonate trial. The increased extracellular buffer capacity and extracellular pH could result in higher [H^+^] gradient across the sarcolemma. This may lead to higher H^+ ^and lactate efflux from working muscles via monocarboxylate co-transporter, a symport carrier of lactate and H^+ ^[30-33].

One of the potential factors that may influence the skilled tennis performance is neural function. It has been shown that central activation failure, changes in neurotransmitter concentrations, inhibition of motoneuron excitability, and disturbance in excitation-contraction coupling may contribute to the development of fatigue in prolonged tennis matches [8]. The central activation deficit of knee extensor muscles occurred progressively during a 3-hour tennis match, indicating a decreasing number of motor units that are voluntarily recruited [3]. Similarly, a decrease in neural drive to the motor unit has also been shown in other types of high-intensity intermittent exercise [34,35]. In tennis, sprints usually occur over very short distances where athletes are unable to reach the maximum speed. Thus, the initial acceleration phase is more important than the maximum speed in the on-court movements [36]. The impairments in neural functions may lead to the slower acceleration in movement and the inability to reach the optimal stroke position. The neural impairments in forearm muscles may also result in the poor control of the racquet. These factors may be partially responsible for the decrease in the skilled performance after the simulated game in our placebo trial, as well as the decreases in ball speed and precision in serve and forehand and backhand strokes after a 2-hr training session [7]. Some evidence suggested that NaHCO_3 _supplementation may alleviate the exercise-induced impairment in the neural functions. NaHCO_3 _supplementation has been shown to increase muscle fiber conduction velocity and reduce force decline in sustained maximal contraction after a 50-min submaximal cycling [22]. An in vitro study also revealed that alkalosis induced by high [HCO3^-^] resulted in an increase in twitch tension in isolated rat phrenic nerve-hemidiaphragm after electrical stimulations [37]. Therefore, it is possible that NaHCO_3 _could help to restore certain level of neural functions after the simulated match, resulting in the better skilled performance in the bicarbonate trial. The effect of NaHCO_3 _supplementation on neural functions requires further research.

It has been argued that intracellular H^+ ^and lactate may not be the major factors in muscular fatigue [38-41]. Similarly, this study showed that NaHCO_3 _supplementation could prevent fatigue-induced decline in performance on the condition of moderate blood [lactate] and unchanged blood pH. The predominant energy source of the short, high-intensity strokes in the Loughborough Tennis Skill Test is phosphocreatine (PCr) because blood [lactate] was only 0.9 ± 0.1 mM after the test [4]. Some studies have proposed that the supplementation of NaHCO_3 _could reduce PCr degradation and increase the power output required to induce the onset of rapid increase in [inorganic phosphate (Pi)]/[PCr] in forearm muscles during incremental wrist-flexion exercise to volitional fatigue [42,43]. However, creatine supplementation had no effect on power and accuracy of tennis strokes in studies of which test protocols were similar to the present study [44,45]. These results suggested that muscle acidosis and creatine content may not be the major factors in the decline in skilled tennis performance as exemplified in this study.

The Loughborough Tennis Skill Test is an optimal method for measuring the fatigue-induced decline in tennis skills as the accuracy of service and groundstroke was significantly declined after volitional fatigue [4]. In addition, the groundstroke accuracy was significantly decreased after the middle of the test [6]. Our results also showed that the consistency of service and forehand ground stroke was impaired after a simulated match in the placebo trial, while it was maintained in the bicarbonate trial.

The current study presented the similar skill level of players to those in the previous studies [4,6]. In Davey *et al*. [4] the average accuracy and consistency scores of service (out of 20) were 4.0 and 9.0, respectively. The average accuracy and consistency scores (out of 20) were 1.5 and 11.3 for forehand ground stroke and 1.8 and 10.4 for backhand ground stroke, respectively. Another study showed a total ground stroke accuracy of 11.8% at the baseline [6]. These indicated that the Loughborough Tennis Skill Test was a suitable measurement for the skills in the present study.

To hit the areas designated for 'accuracy' was a difficult task. The average service accuracy before the simulated match in both trials combined was 18.5% (3.7 out of 20), while the average ground stroke accuracy was 14.5% (5.8 out of 40). It is possible that should the metabolic and/or neural functions be improved, our participants still could not show the improvements in these difficult tasks. Therefore, the improvement may be more apparent in the relatively easier skills such as the consistency.

The absolute intensity of the simulated match used in this study was lower than that in Grand Slam tournaments [2]. This is understandable because our participants were at the national level. Our participants performed 1.67 shots. sec^-1^, compared to approximately 0.75 shots. sec^-1 ^in men's singles in Grand Slams. Each point in our simulated match lasted 10 sec, compared to 4-8 sec in Grand Slams. However, the relative intensity was high. The average heart rate of our participants during the simulated match was approximately 85% of their age-predicted maximal heart rate, similar to 86.2% reported in American Division I collegiate men's singles [29]. It is difficult to design a simulated match that is representative of most real matches as athletes are different in their playing styles, such as baseline or serve and volley. Therefore, the simulated match was designed to include the 3 major types of play, volley, forehand strokes and backhand strokes.

There were several limitations of this study. The content of simulated match was not completely consistent with real tennis matches. The duration of the simulated match was a little shorter than most of the real ones. The psychological strain in real matches was also absent in the simulated match. Secondly, the participants were in free living style between the 2 trials. Although they were asked to maintain their physical activity and dietary patterns before each trial, we could not rule out the possibility that they may not fully comply with the instructions. Thirdly, the participants' motivation to perform with their best effort, including hitting the ball with the maximal power, may also affect the results.

## Conclusions

In conclusion, NaHCO_3 _supplementation could prevent the decline in skilled tennis performance after a simulated match. Future research may include other tennis skills such as volley and drop shot with the measurement of stroke velocity and running speed. The effect of alkalosis on neuromuscular functions and psychological variables such as reactive, anticipatory, and decision-making capacities also warrant further investigation.

## Competing interests

The authors declare that they have no competing interests.

## Authors' contributions

CLW designed the study and assisted the manuscript preparation. MCS carried out blood analysis and assisted the manuscript preparation. CCY assisted the study design and was responsible for conducting the study, including subject recruitment, skill test and data analysis. MHH assisted the design of the study and manuscript preparation. CKC was responsible for statistical analysis and manuscript preparation. All authors have read and approved the final manuscript.
